# Staphylococcal Enterotoxin Gene Cluster: Prediction of Enterotoxin (SEG and SEI) Production and of the Source of Food Poisoning on the Basis of *v*Saβ Typing

**DOI:** 10.1128/AEM.02662-20

**Published:** 2021-02-12

**Authors:** L. Schwendimann, D. Merda, T. Berger, S. Denayer, C. Feraudet-Tarisse, A. J. Kläui, S. Messio, M. Y. Mistou, Y. Nia, J. A. Hennekinne, H. U. Graber

**Affiliations:** a Research Division Food Microbial Systems, Agroscope, Bern, Switzerland; b Laboratory for Food Safety, French Agency for Food, Environmental and Occupational Health & Safety (Anses), Université Paris-Est, Maisons-Alfort, France; c Scientific Service Foodborne Pathogens, Sciensano, Brussels, Belgium; d Université Paris-Saclay, CEA, INRAE, Département Médicaments et Technologies pour la Santé (DMTS), SPI, Gif-sur-Yvette, France; e Université Paris-Saclay, INRAE, MaIAGE, Jouy-en-Josas, France; University of Naples Federico II

**Keywords:** *Staphylococcus aureus*, *egc*, enterotoxin

## Abstract

Currently, only 5 (SEA to SEE) out of 27 known staphylococcal enterotoxins can be analyzed using commercially available kits. Six genes (*seg*, *sei*, *sem*, *sen*, *seo*, and *seu*), encoding putative and undetectable enterotoxins, are located on the enterotoxin gene cluster (*egc*), which is part of the Staphylococcus aureus genomic island *v*Saβ. These enterotoxins have been described as likely being involved in staphylococcal food-poisoning outbreaks. The aim of the present study was to determine if whole-genome data can be used for the prediction of staphylococcal *egc* enterotoxin production, particularly enterotoxin G (SEG) and enterotoxin I (SEI). For this purpose, whole-genome sequences of 75 Staphylococcus aureus strains from different origins (food-poisoning outbreaks, human, and animal) were investigated by applying bioinformatics methods (phylogenetic analysis using the core genome and different alignments). SEG and SEI expression was tested *in vitro* using a sandwich enzyme-linked immunosorbent assay method. Strains could be allocated to 14 different *v*Saβ types, each type being associated with a single clonal complex (CC). In addition, the *v*Saβ type and CC were associated with the origin of the strain (human or cattle derived). The amount of SEG and SEI produced also correlated with the *v*Saβ type and the CC of a strain. The present results show promising indications that the *in vitro* production of SEG and SEI can be predicted based on the *v*Saβ type or CC of a strain.

**IMPORTANCE** Besides having infectious properties in human and animals, S. aureus can produce different enterotoxins in food. The enterotoxins can cause vomiting and diarrhea, often involving many people. Most of these outbreaks remain undiscovered, as detection methods for enterotoxins are only available for a few enterotoxins but not for the more recently discovered enterotoxins G (SEG) and I (SEI). In this study, we show promising results that *in vitro* production of SEG and SEI can be predicted based on the whole-genome sequencing data of a strain. In addition, these data could be used to find the source (human or cattle derived) of an outbreak strain, which is the key for a better understanding of the role SEG and SEI play in foodborne outbreaks caused by S. aureus.

## Abstract (precis)

Besides the infection properties in human and animals, S. aureus can produce different enterotoxins in food. The enterotoxins can cause vomiting and diarrhea, often involving many people.

## INTRODUCTION

Staphylococcus aureus can produce a variety of heat-stable enterotoxins, which, when they are secreted in food, can cause staphylococcal food-poisoning outbreaks (SFPO). According to the European Food Safety Authority (EFSA), staphylococcal enterotoxins (SE) in mixed foods and meat products are among the top 10 pathogen/food vehicle pairs, causing the highest number of hospitalizations in strong-evidence outbreaks. By looking at the number of cases, this trend seems to be on the rise (1). In addition, most of the SFPO are classified as weak-evidence outbreaks, since only the so-called classical enterotoxins (SEA, SEB, SEC, SED, and SEE) can be detected and quantified by commercially available kits (2). Besides these five well-known SE, another 20 have been described recently, and some of them were shown to have an emetic activity (SE) and, hence, could be involved in SFPO (3–9). Enterotoxins for which emetic activity has not yet been proved are considered staphylococcal enterotoxin-like (SE*l*) proteins. As not all SE can be detected directly in food, different methods have been applied in the past to better characterize the S. aureus strains involved in food-poisoning outbreaks, such as pulsed-field gel electrophoresis typing, PCR for detection of the enterotoxin genes, and other methods (10–14). These methods allowed us to evaluate the toxigenic profile of strains or to establish the link between strains and secreted toxins. With the recent advance of whole-genome sequencing (WGS), often each strain involved in an outbreak can be sequenced and characterized genetically, opening new doors to the understanding of the role different SE play in SFPO as well as prediction of antimicrobial resistance and infectivity (15–20).

Twenty years ago, a novel cluster of SE genes, the enterotoxin gene cluster (*egc*), was described containing the so-called new enterotoxins *seg*, *sei*, *sem*, *sen*, *seo*, and *seu* (21, 22). The *egc* is located on the genomic island *v*Saβ and is incorporated in the chromosome as a prophage (16). Literature suggests that about 50% of S. aureus strains harbor an *egc* (21, 23, 24).

For SEG, SEI, SEM, SEN, and SEO, emetic activity has been demonstrated, and it appears that some SFPO might be caused by these enterotoxins (3, 5). A lot is known on the expression of the classical SE (25–27), yet studies on the expression of the new SE are still very limited (28). Genetic backbones and regulatory systems of SE genes vary among S. aureus strains, causing diverse SE expression patterns. Hence, quantities of toxin production vary between strains (25–27).

Due to the lack of information, new methods and tools need to be developed to better understand and predict the expression and regulation mechanisms of the new enterotoxins, including those of the *egc* (29). For this reason, the aim of the present study was to determine whether WGS data can be used to predict staphylococcal enterotoxin production of the *egc in vitro*, particularly of SEG and SEI. These enterotoxins (SEG and SEI) were chosen because they are the only ones (of *egc* enterotoxins) for which a quantitative method for detection is currently available, allowing a direct link for the corresponding WGS data.

## RESULTS

### Strain characterization.

Multilocus sequence typing (MLST) of the 75 S. aureus strains isolated from different sources, like food, humans, animals, and the environment, showed that the most frequently found clonal complexes (CC) are CC5 (*n* = 17), CC20 (*n* = 15), CC30 (*n* = 13), and CC705 (*n* = 11), followed by CC45, CC22, CC50, and CC9 (6, 3, 2, and 2 strains, respectively). In contrast, CC10, CC72, and CC121, as well as an unknown CC, were detected only once (Table 1).

**TABLE 1:** Genotypic characteristics (i.e., clonal complex, enterotoxin genes present on the genome, *v*Saβ type, and *spa* type) and origins of the 75 studied strains^*a*^

Strain	Country	Origin	Source of isolation	CC	Enterotoxin genes	*v*Saβ type	*spa* type
07CEB94STA	Belgium	Food (SFPO)	Ready to eat	5	a, g, i, m, n, o, x	I	t704
11CEB145STA	Japan	Human	Infection	5	a, c, g, i, m, n, o, x	I	*
13CEB178STA	Ireland	SFPO	NA	5	d, j, g, i, m, n, o, r, x	I	t463
13CEB188STA	Ireland	Food (SFPO)	Milk product	5	g, i, m, n, o, x	I	t5829
13CEB191STA	Ireland	Food (SFPO)	Milk product	5	d, g, i, j, m, n, o, r, x	I	t837
13CEB329STA	Belgium	Human (SFPO)	Nose and throat	5	g, i, m, n, o, x	I	t7506
15SBCL1507STA	Ireland	Food (SFPO)	Meat	5	g, i, m, n, o, x	I	*
15SBCL1550STA	Ireland	Food (SFPO)	Ready to eat	5	g, i, m, n, o, x	I	t450
17SBCL08STA	France	Food (SFPO)	Meat	5	g, i, m, n, o, x	I	t111
17SBCL09STA	France	Food (SFPO)	Meat	5	g, i, m, n, o, x	I	t586
17SBCL580STA	Bulgaria	Food (SFPO)	Ready to eat	5	a, d, g, i, j, m, n, o, r, x	I	t535
17SBCL585STA	Bulgaria	Food (SFPO)	Ready to eat	5	a, d, g, i, j, m, n, o, r, x	I	t535
502A	USA	Human	Infection	5	g, i, m, n, o	I	t010
Mu50	Japan	Human	Infection	5	a, c, g, i, l, m, n, o, tst, x	I	t002
N315	Japan	Human	Human faeces	5	c, g, i, l, m, n, o, p, tst, x	I	t002
NZAK3	New Zealand	Human	Skin	5	c, g, i, l, m, n, o, p, x	I	t002
ST288	England	Human	Urine	5	g, i, m, n, o	I	t1003
18SBCL679	Switzerland	Food (SFPO)	Milk product	9	g, i, m, n, o, u, x, y, 27	XIII	t899
G19F	Italy	Animal	Mastitis (cow)	9	g, i, m, n, o, u	XIII	t100
13CEB177STA	Ireland	NA	FPO	10	c, g, i, m, n, o, u, x	XVII	t148
11CEB277STA	Italy	Food	Milk product	20	g, i, m, n, o, u, x	XII	t3929
11CEB279STA	Italy	environment	NA	20	g, i, m, n, o, u, x, y	XII	t325
15SBCL1292STA	France	Food (SFPO)	Milk product	20	g, i, m, n, o, u, x	XII	*
15SBCL1299STA	France	Food (SFPO)	Ready to eat	20	g, i, m, n, o, u. x. y	XII	t164
15SBCL1397STA	France	Food (SFPO)	Milk product	20	g, i, m, n, o, tst, u, x	XII	t164
15SBCL1409STA	France	Food (SFPO)	Milk product	20	g, i, m, n, o, u, x, y	XII	*
15SBCL1428STA	France	Food (SFPO)	Milk product	20	g, i, m, n, o, u, x, y	XII	*
17SBCL202STA	France	Food (SFPO)	Milk product	20	g, i, m, n, o, u, x, y	XII	t164
17SBCL208STA	France	Food (SFPO)	Milk product	20	g, i, m, n, o, u, x, y	XII	t458
17SBCL214STA	France	Food (SFPO)	Milk product	20	g, i, m, n, o, u, x, y	XII	*
17SBCL220STA	France	Food (SFPO)	Milk product	20	g, i, m, n, o, u, x, y	XII	t10134
17SBCL225STA	France	Food (SFPO)	Milk product	20	g, i, m, n, o, u, x, y	XII	*
18 SBCL 680	Switzerland	Food	Milk product	20	g, i, m, n, o, u, x, y	XII	t1544
18 SBCL667	Switzerland	Food	Milk product	20	g, i, m, n, o, u, x, y	XII	*
G11F	Switzerland	Animal	Mastitis (cow)	20	g, i, m, n, o, u	XII	t2736
13CEB179STA	Ireland	NA	FPO	22	c, g, i, m, n, o, u, x	XVI	*
15SBCL1517STA	Ireland	Food (SFPO)	Meat	22	c, g, i, l, m, n, o, u, x	XVI	t645
15SBCL1527STA	Ireland	Food (SFPO)	Ready to eat	22	g, i, m, n, o, u, x	XVI	*
13CEB181STA	Ireland	Food (SFPO)	Ready to eat	30	a, g, i, m, n, o, u	III	t3018
13CEB312STA	Belgium	Food (SFPO)	Ready to eat	30	a, g, i, m, n, o, u	III	t022
13CEB313STA	Belgium	Human (SFPO)	Human faeces	30	a, g, i, m, n, o, u	III	*
13CEB317STA	Belgium	Human (SFPO)	Nose and throat	30	a, g, i, m, n, o, u	III	*
13CEB318STA	Belgium	Human (SFPO)	Nose and throat	30	a, g, i, m, n, o, u	III	*
13CEB327STA	Belgium	Human (SFPO)	Nose and throat	30	g, i, m, n, o, u	III	*
13CEB328STA	Belgium	Human (SFPO)	Nose and throat	30	g, i, m, n, o, u	III	*
18 SBCL671	Switzerland	Food (SFPO)	Milk product	30	g, i, m, n, o, tst, u	III	t021
18SBCL675	Switzerland	Food	Ready to eat	30	g, i, m, n, o, tst, u	III	t021
18SBCL678	Switzerland	Food	Ready to eat	30	g, i, m, n, o, u	III	t166
ATCC 25923	USA	Human	Skin	30	g, i, m, n, o, u	III	t021
KS90	Switzerland	Food (SFPO)	Ready to eat	30	g, i, m, n, o, u	III	t021
MRSA252	USA	Human	Infection	30	g, i, m, n, o, u	III	t018
07CEB90STA	Belgium	Food (SFPO)	Ready to eat	45	c, g, i, m, n, o, u	XXII	t1040
18 SBCL 676	Switzerland	Food	Ready to eat	45	g, i, l, m, n, o, u	XXII	t505
18SBCL673	Switzerland	Food (SFPO)	Milk product	45	g, i, m, n, o, u	XXII	t015
18SBCL674	Switzerland	Food (SFPO)	Milk product	45	g, i, m, n, o, u	XXII	t015
18SBCL677	Switzerland	Food	Ready to eat	45	g, i, l, m, n, o, u	XXII	t505
USA600	USA	Human	Infection	45	g, i, m, n, o, u	XXII	t004
18SBCL672	Switzerland	Food	Milk product	50	i, m, n, o, u, x, z	XXI	t246
GN3	Japan	Human	NA	50	i, m, n, o, u	XXI	t185
13CEB323STA	Belgium	Human (SFPO)	Nose and throat	72	c, x, g, i, m, n, o, u	XX	t022
05CEB52STA	NA	Human	FPO	121	b, g, i, m, n, o, u, y, x	XIX	*
18SBCL669	Switzerland	Food	Milk product	479	d, g, i, m, n, o, u, x	XI	t7013
G68P	Switzerland	Animal	Mastitis (cow)	479	g, i, m, n, o, u	XI	t7013
13CEB182STA	Ireland	Food (SFPO)	Milk product	705	c, i, m, n, o, tst, u, x	IV	t529
13CEB190STA	Ireland	Food (SFPO)	Milk product	705	c, i, m n, o, tst, u, x	IV	t529
15SBCL1438STA	France	Food (SFPO)	Milk product	705	c, i, m n, o, tst, u, y, x	IV	t529
18SBCL670	Switzerland	Food	Milk product	705	c, i, m n, o, tst, u, y, x	IV	t529
M1280	Switzerland	Animal	Mastitis (cow)	705	c, i, m, n, o, u	IV	t529
M1655	Switzerland	Animal	Mastitis (cow)	705	c, i, m, n, o, u	IV	t529
M2323	Switzerland	Animal	Mastitis (cow)	705	c, l, i, m n, o, tst, u	IV	t529
M2682	Switzerland	Animal	Mastitis (cow)	705	c, i, m, n, o, u	IV	t529
M2839	Switzerland	Animal	Mastitis (cow)	705	c, l, i, n, o, tst, u	IV	t529
M3783	Switzerland	Animal	Mastitis (cow)	705	i, m, n, o, u	IV	t529
RF122	Ireland	Animal	Mastitis (cow)	705	c, i, l, m, n, o, u, tst, x, y, z	IV	t529
17SBCL13STA	France	Food (SFPO)	Meat	**	a, g, i, m, n, o, x	XVIII	t13785

a NA, data not available; SFPO, food poisoning outbreak; *, unknown *spa* type; **, unknown clonal complex (CC).

The strains from the most frequently found CCs (CC5 and CC30) originated from a vast geographical range and were isolated from either human or food. In contrast, the CC20 and CC705 strains, always originating from France, Italy, and Switzerland, were isolated either from dairy products or bovine mastitis (Table 1).

*spa* typing of the 75 strains revealed that in most cases the strains belonging to a single CC were allocated to different *spa* types. Perfect agreement between CC and *spa* type was found only for CC705 (*n* = 11), where all strains were allocated to t529. For 15 strains, *spa* typing resulted in an unknown type, of which the majority belonged to CC30 and CC20 (5 and 6 unknown *spa* types, respectively).

Besides *egc*, the 75 strains also harbored other non-*egc* SE genes (Table 1). Indeed, from genome assembly, all 27 SE genes were detected in one of the strains at least once, yet it is noteworthy that the five strains belonging to CC5 often carried additional SE genes, such as *selx* (in 4 strains), *sea* (in 3 strains), and a plasmid containing *sed*, *sej*, and *ser* (in 2 strains). Furthermore, CC30 (*n* = 13) harbored *sea* in 6 strains and *tst* (toxic-shock toxin) in 2 strains.

CC705 was comprised of *sec*, *tst*, *selx*, and *sel*, whereas CC20 often carried *selx* and *sey* (in 14 and 11 out of 15 strains, respectively).

### Allocation of the strains to their *v*Sa**β** types and diversity of SEG and SEI.

**FIG 1:**
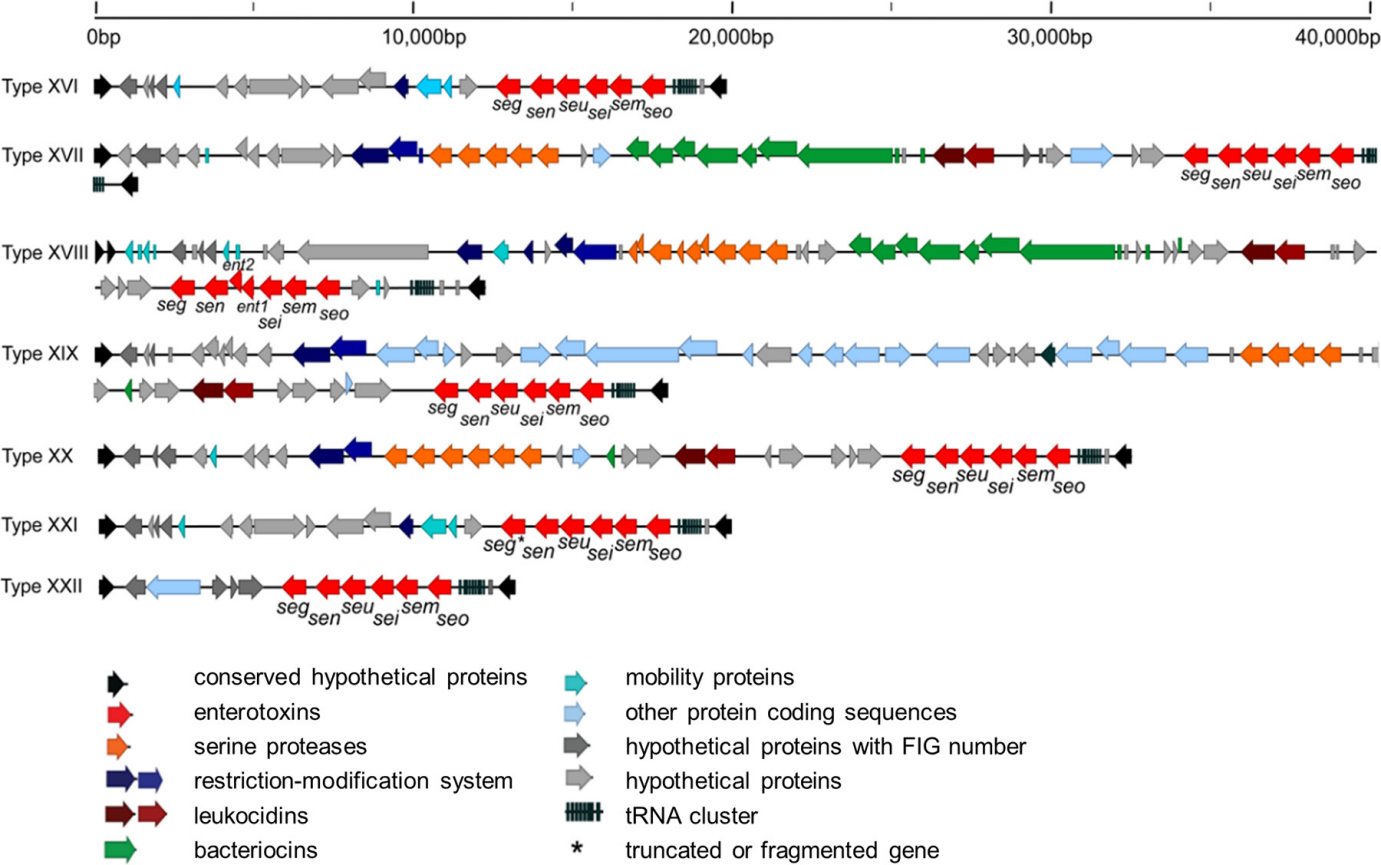
Representation of the newly defined S. aureus genomic island *v*Saβ types XVI to XXII. The virulence-associated genes, and other hypothetical genes located on *v*Saβ, are also presented. For each *v*Saβ type, one reference strain is shown. Arrows show the orientation of open reading frames. FIG numbers are *hp* genes that were assigned to a FIG number by the RAST (Rapid Annotations using Subsystem Technology) pipeline. *ent1* and *ent2* of *v*Saβ type XVIII are genes that were already described by Collery and Smyth (78). *, truncated or fragmented gene.

In 59 of 75 strains (79%), the *v*Saβ type could be allocated to an existing one with overall similarities of >90%. For the remaining 16 strains, new *v*Saβ types were defined by numbering continuously from XVI onward (Fig. 1), resulting in seven new *v*Saβ types (XVI to XXII). Three strains were allocated to *v*Saβ type XVI, two strains to *v*Saβ type XXI, and six strains to *v*Saβ type XXII, respectively (Table 1). For the remaining *v*Saβ types (XVII, XVIII, XIX, and XX), only one strain of each was found.

The seven newly defined *v*Saβ types (Fig. 1) all contained, in addition to the *egc* genes, virulence-associated and hypothetical genes. *v*Saβ types XVII and XVIII carry bacteriocins and serine proteases, whereas *v*Saβ type XIX was notably (approximately 20,000 bp) longer than the other *v*Saβ types and carried numerous genes coding for hypothetical proteins. *v*Saβ type XXII was shorter than all other *v*Saβ types (approximately 13,000 bp) and did not carry any additional virulence-associated genes besides the *egc* genes.

Within each *v*Saβ type, an amino acid identity of 100% for each SE was observed. However, SE differences were observed among different *v*Saβ types (Table 2). Among all strains included in the study, the SEG amino acid similarity varied between 96% and 100%, with a maximum of 9 amino acids of difference, compared to strain Mu50 (reference). For SEI, the similarity varied between 93% and 100%, with a maximum difference of 19 amino acids.

**TABLE 2:** Amino acid similarity of SEG and SEI compared to the reference strains^*a*^ (Mu50 and *v*Saβ type I)

*v*Saβ type	Amino acid similarity (%)	
	SEG	SEI
I	100^R^	100^R^
III	97	95
IV	*	95
XI	97	93
XII	100	100
XIII	99	100
XVI	100	99
XVII	96	97
XVIII	100	100
XIX	97	93
XX	99	100
XXI	*	97
XXII	100	99

a Each *v*Saβ type sequence is represented based on 100% intergroup similarity. Superscript R, reference; *, gene absent.

### Phylogenetic analysis of the core genome.

**FIG 2:**
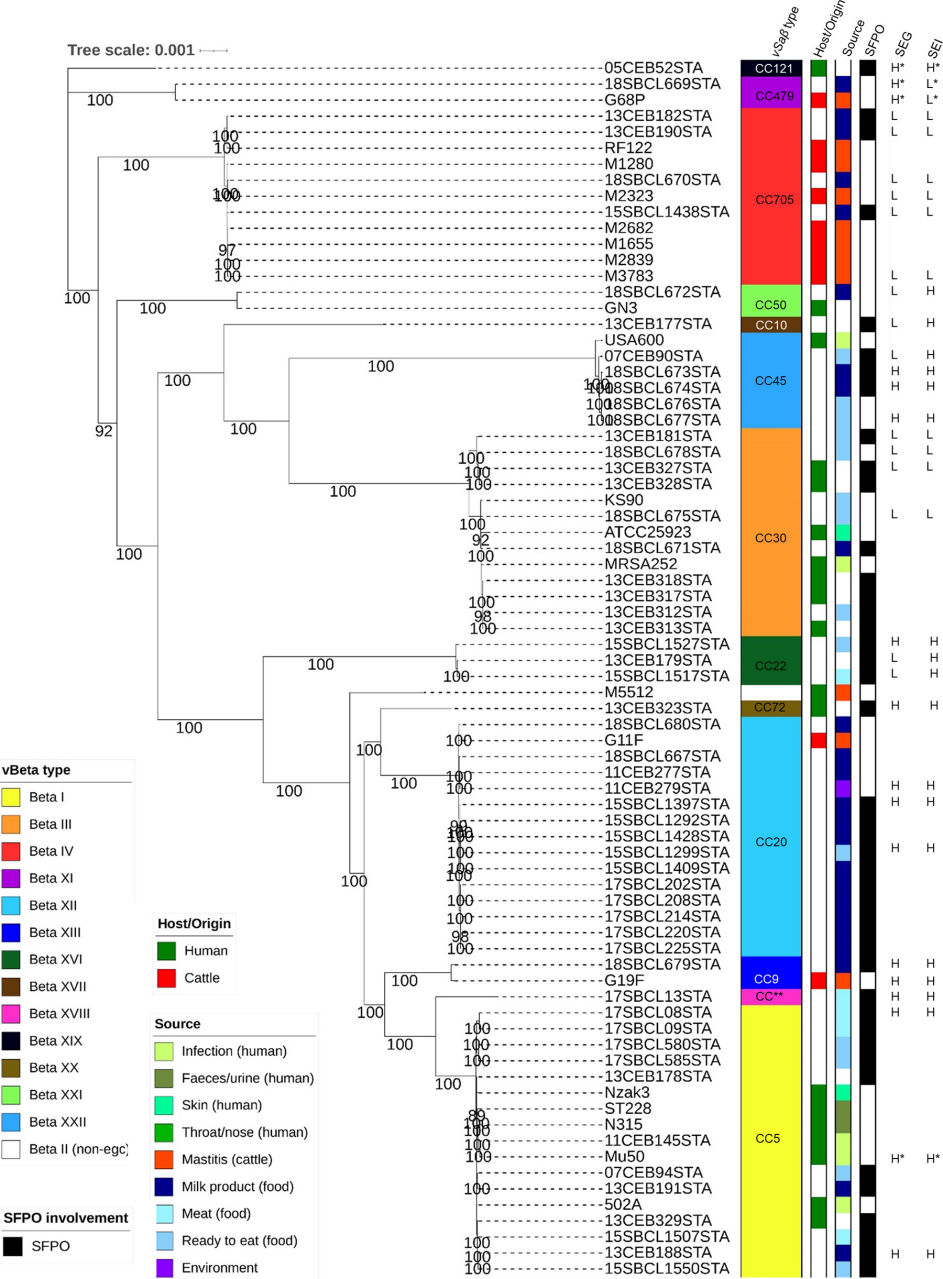
Maximum likelihood phylogenetic tree based on the core genome (nucleotidic sequences) showing the evolutionary relationship among 75 isolates of Staphylococcus aureus (all strains positive for the enterotoxin gene cluster) recovered from human, animal, environment, and food samples (left). At the right, for each strain its clonal complex (CC), origin of the strain, source of the strain, and involvement in staphylococcal food poisoning outbreak (SFPO) is given. Bootstrap values of >80 are shown. Production of enterotoxin G (SEG) and I (SEI) for the 32 analyzed strains is also given (last two columns). These are shown as L for low enterotoxin production and H for high enterotoxin production. *, statistical outliers; **, unknown CC.

To evaluate the evolutionary relationship of S. aureus strains included in the present study, their phylogeny was evaluated based on their core genomes. The tree shows a perfect concordance between the phylogenetic clades, CCs, and *v*Saβ type of the strain (Fig. 2). For *v*Saβ type IV, XI, XII, and XIII, a perfect concordance was observed between strains isolated from milk products, and animal mastitis can be observed (no human strains harbored these *v*Saβ types). On the other side, strains harboring *v*Saβ type I, III, and XXII were only found in humans (including infections) and food isolates. No animal strains harbored these *v*Saβ types. SFPO strains were found in every *v*Saβ type.

### Enterotoxin production.

**FIG 3:**
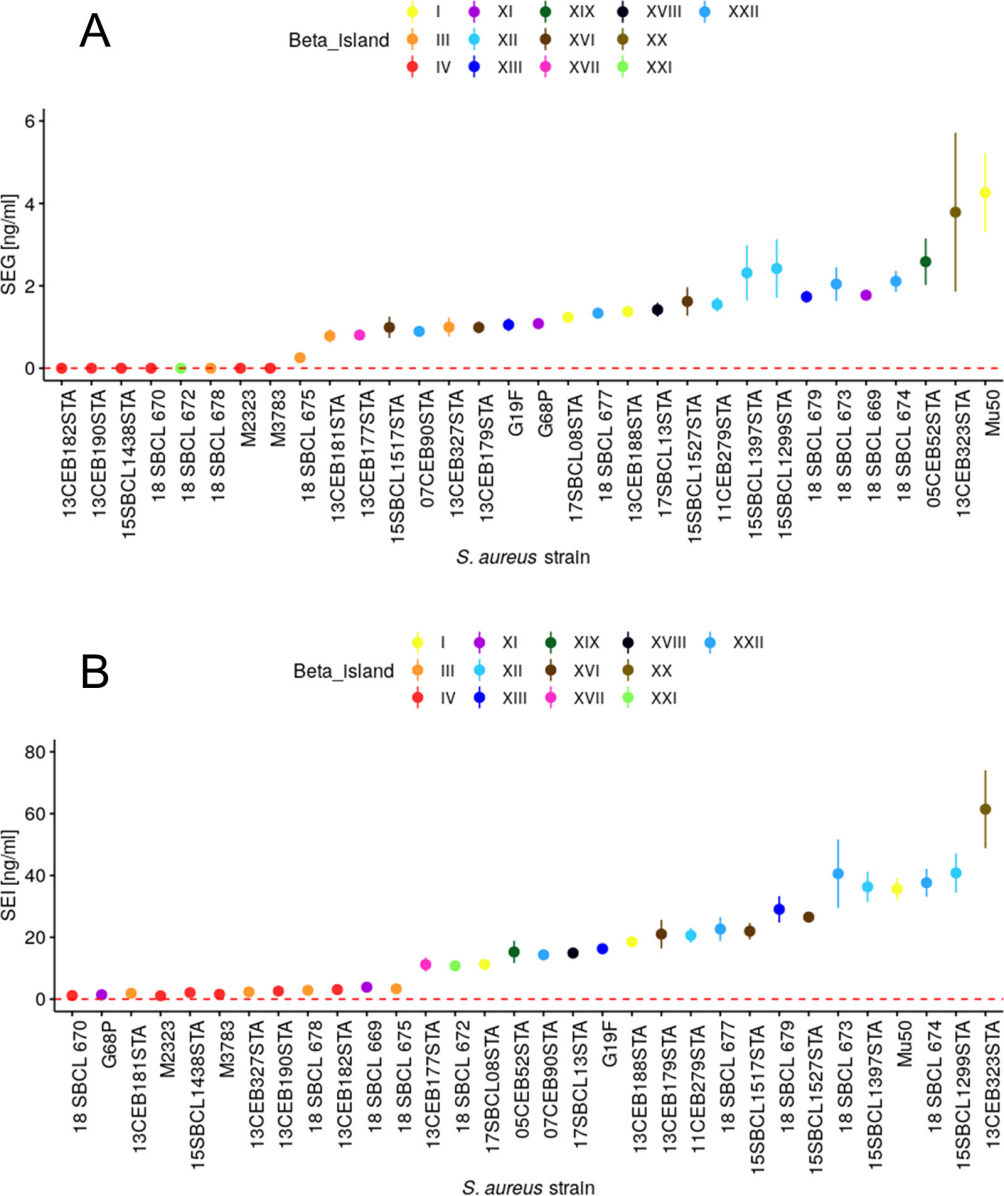
Amount (nanograms per milliliter) of staphylococcal enterotoxins G and I (A and B, respectively), measured with enzyme-linked immunosorbent assay. Each point is the average measurement from three biological replicates, and the corresponding bars represent standard deviations. Strains were incubated in brain heart infusion (BHI) for 24 h at 37°C. The limit of detection of the corresponding enterotoxin is presented by red dashed line (LOD SEG, 0.001 ng/ml; LOD SEI, 0.037 ng/ml).

SEG production ranged from below the limit of detection (LOD; 0.001 ng/ml) to 4.26 ± 0.78 ng/ml, with a median of 1.17 ng/ml. SEG production below the LOD (0.001 ng/ml) was observed for *v*Saβ IV and XXI. One strain carrying *v*Saβ III (18SBCL675) showed nondetectable quantities of SEG, whereas the other two strains harboring *v*Saβ III had values between 0.26 ± 0.01 and 0.78 ± 0.13 ng/ml. All the other strains showed values between 0.80 ± 0.11 and 4.26 ± 0.78 g/ml. By visual data inspection (Fig. 3A), two levels of SEG production can be distinguished: 9 strains that generated low (L) and 23 strains that produced high (H) concentrations of SEG. The median concentration for the L producer was 0 ng/ml (minimum [min], 0 ng/ml; maximum [max], 0.26 ± 0.01 ng/ml) and for the H producer was 1.42 ± 0.14 ng/ml (min, 0.783 ± 0.13 ng/ml; max, 4.26 ± 0.78 ng/ml). The difference between medians was highly significantly (*P < *0.001).

The amount of SEI produced (LOD, 0.037 ng/ml) by the strains ranged from 1.06 ± 0.17 ng/ml to 61.43 ± 10.29 ng/ml (median, 14.31 ng/ml) (Table 1 and Fig. 3B). According to their SEI production, strains could again be visually allocated to two different levels, L producers (producing 1.06 ± 0.17 to 3.85 ± 0.99 ng/ml; median, 2.22 ng/ml) and H producers (10.77 ± 1.22 to 61.43 ± 10.29 ng/ml; median, 21.51 ng/ml). The L strains belonged to the *v*Saβ types III, IV, and XI, whereas the H strains belonged to *v*Saβ types I, XII, XIII, XVI, XVII, XVIII, XIX, XX, XXI, and XXII (*P < *0.001 between L and H).

To assess a possible relationship between SEG and SEI production, first a robust linear regression (see Fig. S1 and S2 in the supplemental material) was performed, identifying four outliers (G68P, 18SBCL669, Mu50, and 05CEB52). These outliers were not taken into consideration for a second, ordinary least-square linear regression analysis (Fig. S3). This regression was modeled to [SEI] = 15.49 × [SEG] + 0.63, with *R* = 0.940 (*P* < 0.001), where brackets indicate the SE concentrations in nanograms per milliliter.

## DISCUSSION

In the present study, we demonstrate that SEG and SEI production *in vitro* can be predicted using genomic data. In fact, there are strong indications that the amount these SE produced depends on the *v*Saβ type. Furthermore, with the analysis and findings described here, it is now possible to infer the origin of an *egc*-containing S. aureus strain (human derived, cattle derived) that is involved in an SFPO. As the *v*Saβ type is perfectly linked to the CC of a strain, as shown in the present study and in a previous report from Kläui et al. (30), the SE production and the origin of the SFPO also can be predicted based on the CC of the strain obtained by MLST, a typing method that is well established.

Previous studies already demonstrated that different strains can produce different amounts of SE, but in most cases the link to the genome was missing (31, 32).

In this study, the focus was on the *egc* enterotoxins that, according to previous studies (3, 12, 33, 34), are harbored by about 50% of S. aureus strains. The importance of the *egc* enterotoxins regarding food safety has been shown by Johler et al. (3), who described the probable *egc* enterotoxins’ involvement in foodborne outbreaks. However, strong evidence could not be confirmed, as the enterotoxin measurement in the food and from the bacteria could not be performed due to lack of appropriate methods. This could also be the reason why a lot of *egc*-caused SFPO remain undiscovered. In this study, for two enterotoxins (SEG and SEI) out of the five *egc* enterotoxins, an enzyme-linked immunosorbent assay (ELISA) method was available, whereas for the other *egc* enterotoxins this still is not the case. *seu* was not considered at all, as there is no literature demonstrating its emetic activity. Due to this lack of information about *egc* enterotoxins, new methods and tools need to be developed to better understand and predict their expression and regulation mechanisms (29). As a consequence, the aim of the present study was to determine whether WGS data can be used to predict staphylococcal enterotoxin production of the *egc in vitro*, particularly of SEG and SEI.

### Prediction of SEG and SEI production *in vitro*.

For the present study, 75 strains were chosen, originating from both human hosts and animal (cattle) as well as from environmental and food sources, with special attention on SFPO strains (35). Out of the 75 strains, 60 were allocated to the 15 previously defined *v*Saβ types (30). The remaining 15 strains could be grouped into 7 newly defined *v*Saβ types (Fig. 1). According to these new insights, using the *v*Saβ types seems to be a very precise tool to characterize the different *egc* present in S. aureus strains instead of using *egc* types I to VI, as has been described previously (14, 21, 22, 36, 37).

The present study shows that there are two groups of SE producers, strains that produce low levels of SEG and SEI and strains with increased SE production (for both, SE *P < *0.001). A special case is the absence of SEG production for *v*Saβ IV and XXI. This is explained by the fact that both had a truncated *seg* gene, resulting in an incomplete, nondetectable protein.

A very high linear dependency was observed between the production of SEG and SEI (*R* = 0.98, *P < *0.001), while the amount of SEI measured was approximately 16 times higher than that of SEG. The high correlation between SEG and SEI production suggests that both SE are regulated primarily by the same transcription factor as that proposed by Kusch et al. (38). This hypothesis, however, neglects the fact that the SEG production is 16× lower than that for SEI, accounting for a fine tuning by additional transcription factors, as observed for other SE (38, 39).

During the first robust linear regression analysis, outliers were observed (G68P, 18SBCL669, and 05CEB52). For these strains, all members of *v*Saβ types XI and XIX, the production of SEI was always lower than SEG production (see Fig. S2 in the supplemental material). As demonstrated in Table 2, SEI of both *v*Saβ types showed the lowest similarity (93%) compared to the reference (Mu50). These findings indicate that the monoclonal antibody used for the present study matches incompletely with the SEI epitopes produced by *v*Saβ type XI- and XIX-producing strains, resulting in a reduced detection of SEI quantities. Besides the technical aspect, it cannot be ruled out; however, regulation of SEI production is special for these *v*Saβ types. To clarify these ambiguities, additional studies are required.

The results of the present European study were not in agreement with the results published by Omoe et al. (40), who detected SEI in only 40% of the strains and SEG was not detected at all. In our study, SEG was produced by 96% of the strains and SEI for 100% of the strains, being positive for the two enterotoxin genes detected by NAuRa (35). Only for one strain (18SBCL678) was *seg* predicted, but SEG enterotoxin was not detected. As our results were generated from a large variety of strains, the involvement of the *egc* enterotoxins in SFPO should be reconsidered.

### Inferring the origin of an SFPO-involved strain.

Looking at the major *v*Saβ types found in this study (I, III, IV, XII, and XXII), it was observed that in each group there are SFPO-associated strains (isolated from food) and strains that are human (infection) or cattle (mastitis) derived but never both for the same *v*Saβ type.

In addition to our previous study with 15 allocated *v*Saβ type observed (30), we found 9 new types. Again, a perfect concordance between *v*Saβ type and CC was found, confirming this observation as a general principle in S. aureus. This principle can now be applied for evaluation of *egc*-containing strains involved in SFPO. In fact, instead of inferring the *v*Saβ type involved in the SFPO, the common and simpler method of CC assessment can be performed. This is particularly easy for WGS data, as the reads can be directly uploaded to an Internet app, such as cge.cbs.dtu.dk, for inferring of the sequence type (ST), which is then used together with the pubMLST database program (41) to obtain the corresponding CC. If WGS data are not available, the standard MLST procedure can be performed using standard PCR and Sanger sequencing for the seven housekeeping genes (*arcC*, *aroE*, *glpF*, *gmk*, *pta*, *tpiA*, and *yqiL*) (42). Instead of the original primers (35), the newly designed primer by Boss et al. (43) can be applied. They enable unidirectional Sanger sequencing, which considerably saves cost, work, and time.

The suspected reason for this strong link between CC and *v*Saβ type is that *v*Saβ acquisition by horizontal transfer in the S. aureus genome happened immediately before or simultaneously to clonal diversification of S. aureus (30). This hypothesis is also supported by the phylogenetic analysis of the core genomes of the present study (Fig. 2), showing a perfect agreement between the phylogenetic clade, CC, and *v*Saβ type.

The CC can be used to perform an association of an *egc*-carrying SFPO strain to a specific origin (human or cattle). As can be seen in Fig. 2, CC705 and CC20 are strains strictly associated with bovine mastitis and dairy products. In fact, CC705-positive strains are classical pathogens of bovine mastitis observed in- and outside of Europe (43–45). In addition, they are also frequently present in delivered milk (43) and cheese (46). CC705 strains are uniquely positive for *spa* type t529 (Table 1) and are typical colonizers of bovine skin as well as infections of bovine wounds (47). Similar findings are also true for CC20-positive strains. These can also cause bovine mastitis and are present in delivered milk, but they are less abundant than CC705 (43).

On the other hand, strains allocated to CC5, CC30, and CC45 were exclusively isolated from human samples (infection, skin, feces, nose, and throat) and from food (Fig. 2), where human handling was very likely (ready-to-eat products). Furthermore, these CCs are widely described in the literature as being found in human infections (48–51). This is a further advantage of CC nomenclature as literature about them is broad (23, 52, 53), enabling us to extend the scope beyond an *egc* enterotoxin-caused SFPO.

### Application of new insights in evaluation of *egc*-caused SFPO.

The involvement of *egc* enterotoxins in foodborne outbreaks is highlighted by the fact that *v*Saβ types (and the corresponding CC) from S. aureus strains producing high levels of SEG and SEI are also described to be involved in foodborne outbreaks, especially CC5, CC20, and CC45 (23, 34, 53). Furthermore, certain strains of CC45 (harboring *egc*) do not harbor any classical enterotoxin (34, 54), yet these strains could have been involved in foodborne outbreaks.

As an example, we deal with strain 18SBCL673, which was involved in a foodborne outbreak related to artisanal goat cheese in southern Switzerland (54) and was included in the present study. It is characterized by the presence of just *egc* enterotoxins, as shown by NAuRa, and produces a substantial amount of SEG (2.04 ± 0.33 ng/ml) and SEI (40.58 ± 9.03 ng/ml). It is positive to *v*Saβ type XXII and CC45. As the strain had been isolated from goat cheese, it could be hypothesized that goat milk was the probable source. However, according to the present study (Fig. 2), it is clear that the origin of the involved strain is, with a high probability, human. As a consequence, the SFPO caused by this strain was a highly human contamination during cheese manufacturing. This conclusion is supported by the fact that CC45 is never found in goats and goat milk (55, 56).

### Conclusions.

The presented study demonstrates that the *in vitro* production of SEG and SEI can be predicted based on the *v*Saβ type and the CC of a strain. Furthermore, the *v*Saβ type/CC enables us to predict the source of an *egc*-positive SFPO strain (animal or human derived). Due to the perfect correlation between CC and *v*Saβ type, the use of common CC typing is an easy and quick way to characterize a strain involved in an SFPO. Therefore, it is a good alternative to the proposed *egc* typing (I to IV), a method that results in only four biologically irrelevant types.

This information will enhance the ability to better understand the involvement of the *egc* enterotoxins in SFPO. The fact that the *egc* is found in more than 50% of the S. aureus strains and, according to our study, exactly 75% expressed SEG and 100% expressed SEI are further indications that these and other *egc* enterotoxins are involved in SFPO.

## MATERIALS AND METHODS

### Bacterial strain and genome collection.

The general aim was to use *egc*-harboring S. aureus strains representing a large diversity in their genomes and origins. To achieve this, 75 strains and genomes from different sources (food, environment, animal, and human) as well as different geographical origins were chosen (Table 1). SFPO genome sequences and strains (42 genomes and strains) were obtained from the collection of the European National Reference Laboratory for Coagulase-Positive Staphylococci (EURL CPS; Maisons-Alfort, France). Nine Swiss bovine mastitis strains were used from the Agroscope strain and genome collection; these strains were sampled previously by Fournier et al. (57) and their genome sequenced by Kläui et al. (30). For genomic and phylogenetic analysis, seven strains of human and animal origins were obtained from NCBI (reference sequence database; https://www.ncbi.nlm.nih.gov) to increase the sample size and variation of the strains. Two strains (Mu50 and N315) were obtained from P. Moreillon (University of Lausanne). Thirteen *egc*-containing strains were isolated from food in Switzerland (details are described below). An overview of the bacterial strain collection used in study is listed in Table 1.

### Characterization of Swiss food strains.

Forty-five Swiss S. aureus strains originating from food were obtained from the Federal Food Safety and Veterinary Office (kindly provided by A. Baumgartner). The presence of *egc* genes in these strains was determined by applying a real-time PCR assay with melting curve analysis (mPCR) for detection of *seg*, *sei*, *sem*, *sen*, and *seo*. For detection of *seg* and *sei*, primers and PCR conditions were applied as described by Cosandey et al. (58). For detection of *sem*, *sen*, and *seo*, new primers were designed (Table 3). The detection of *seu* was omitted, as its emetic activity has not been shown so far. After being cultured at 37°C for 24 h on blood agar (bioMérieux Suisse s.a., Geneva, Switzerland), DNA was extracted from single colonies of S. aureus. A colony was picked and resuspended in 100 μl of 10 mM Tris-HCl and 10 mM EDTA (pH  8.5), incubated at 95°C for 10 min, and immediately stored on ice. The lysates were diluted 1:100 in H_2_O to be used as templates for the different mPCRs (43). For all mPCRs, the total volume was 20 μl, containing 300 nM corresponding forward and reverse primer (Table 3), 1× Kapa Sybr Fast (Kapa Biosystems Inc., Woburn, MA), and 2.5 μl of 1:100 diluted DNA template. The mPCR run began with an initial denaturation at 95°C for 3 min, followed by 35 cycles of denaturation at 95°C for 3 s, annealing and extension in a single step at 60°C for 50 s, and a final extension step at 60°C for 5 min. Melting of the amplicons was performed between 60°C and 94°C, with increments of 1°C and a 5-s waiting time at each step. The mPCRs were performed using a Rotor-Gene 6000 real-time thermal cycler (Corbett Life Science, Mortlake, Australia).

**TABLE 3:** Primers for detection of enterotoxin genes developed and used in this study

Gene	Primer^*a*^	Sequence 5′–3′	Amplicon size (bp)
*sem*	Gsem_S	GATGTCGGAGTTTTGAATCTTA	584
	Gsem_AS	ACTTTCAGCTTGCCCTGTT	
*sen*	Gsen_S	TTCTTCCAGTTAAGCCTACACA	218
	Gsen_AS	CTGATATAACGTGGCAATTAG	
*seo*	Gseo_S	TAAAGCGCATTGTCATGGTGAG	348
	Gseo_AS	ACATCACTAGTCATTCGGTCATA	

a S, sense primers; AS, antisense primers.

Primer specificity (Table 3) was tested with S. aureus strains that were previously sequenced, namely, G11F, G19P, M1280, M1655, M2323, M2682, M3783, Mu50, and N315 (Table 1).

Applying the mPCR for detection of the *egc* genes showed that only 40% of the strains were *egc* positive. Based on the diversity of their origins, 14 *egc*-positive strains were selected.

These Swiss strains, isolated from food, were sequenced as follows. Strains were cultured at 37°C for 24 h on blood agar, 3 to 4 colonies were suspended in 4.5 ml tryptic soy broth (TSB; Becton, Dickinson), and incubated 18 h (37°C, with shaking). From this overnight culture (ONC), 1 ml was suspended in 500 ml TSB and incubated under the same conditions. The resulting ONC was centrifuged for 23 min (7°C, 6,000 × *g*) (Cellsep 6/720R; Henderson Biomedical Ltd., Lower Sydenham, UK). The supernatant was discarded and the pellet resuspended in 15 ml 10 mM Tris-HCl, pH 7.8, and transferred to a falcon tube, which again was centrifuged for 5 min (4°C, 18,000 × *g*). After centrifugation, the pellet was treated using the NucleoBond Xtra Maxi kit (Machery Nagel, Düren, Germany) according to the manufacturer’s protocol, with the following modifications: instead of resuspending the pellet directly in 24 ml RES (from the kit), the pellet was resuspended in 2 ml RES containing 350 mg glass beads (425 to 600 μm; Merck, Darmstadt, Germany) and shaken on a Bead Ruptor at level 6 for 45 s (Bead Ruptor Elite; Omni International, Kennesaw, GA, USA). After centrifugation for 5 min (4°C, 13,500 × *g*), 22 ml was added to the supernatant, and DNA was then extracted according to the protocol of the manufacturer of the kit. The pellet was resuspended in 200 μl ddH_2_O (double-distilled water) and further purified by applying the High Pure PCR template preparation kit protocol (Roche, Basel, Switzerland). DNA quality was considered sufficient if the optical density at 260 nm (OD_260_)/OD_280_ was ≥1.8 and OD_260_/OD_230_ was ≥1.9 (measured with a QuickDrop spectrophotometer; Molecular Devices, San Jose, CA). The extracted DNA (representing the whole genome) was sequenced by an Illumina HiSeq at Eurofins GATC (Constance, Germany), generating more than 1.5 Gb of reads.

### Bioinformatics.

The reads from the strains from EURL CPS were obtained from the European Nucleotide Archive database (https://www.ebi.ac.uk/ena). For these reads and the reads from the Swiss food strains, the method for assembly and annotation was applied according to Merda et al. (35). Before the assembly, reads were normalized using BBnorm (https://jgi.doe.gov/data-and-tools/bbtools/) to have a maximum coverage of 100×. Normalized reads were trimmed using Trimmomatic (59). Quality filtering then was performed, removing reads shorter than 50 bp as well as excluding bases having a Phred quality score lower than 30. With these filtered reads, assembly was performed in three steps: (i) a *de novo* assembly was generated using SPAdes (v.3.9.1) (60) applying the default parameters, (ii) scaffolding was performed in MeDuSa (61), using the nearest complete public genome of S. aureus estimated by Mash (62), and (iii) gaps were closed using GMcloser (63). The quality of each assembled genome was assessed with QUAST (v.4.3) (64). The assemblies were annotated using Prokka (v.1.11) (65) and RAST (66) for the prediction of coding sequences (CDSs).

### MLST, *spa* type, and *v*Sa**β** type allocation.

For all 75 genomes used in this study, three typing methods were applied to further characterize the strains genomically: (i) multilocus sequence typing (MLST), (ii) *spa* typing, and (iii) *v*Saβ typing. The MLST of the seven housekeeping genes (67) and *spa* typing (68) were done by using the Center for Genomic Epidemiology online platform (http://www.genomicepidemiology.org/). In the pubMLST database program (41), the sequence types (STs) from MLST were used to allocate each strain to a CC. For ST504 in the actual pubMLST database, no corresponding CC is available; as a consequence, this ST was allocated to CC705, as also described in the literature (43). *v*Saβ islands were identified in the genome by applying the method described by Kläui et al. (30). Briefly, if the *v*Saβ island of a strain had a sequence similarity of ≥90% to the reference strain of any existing *v*Saβ type, it was considered of the same type (30). If the sequence similarity was <90%, the *v*Saβ island was defined as a new type. All alignments were performed by using the Needleman-Wunsch algorithm of Clone Manager Professional 9 software (Scientific & Educational Software, Denver, CO).

### Enterotoxin gene profiles.

The enterotoxin gene profiles of the S. aureus strains, based on the WGS, were determined using the NAuRA tool (https://github.com/afelten-Anses/NAuRA). The screening of the enterotoxins was performed using the gene sequence and their relative protein sequence of the already described 27 SE and the estimated parameters of BLAST by Merda et al. (35).

### Phylogenetic analysis.

The core genome of each of the 75 strains was determined by the Roary pipeline (69). For this, the previously obtained GFF3 file from Prokka was used as an input containing all of the strains’ genes as detected by the software. All genes of a strains’ core genome were then concatenated. A multiple-sequence alignment (MSA) (using MAFFT [70]) was performed using the concatenated core genomes of all the strains. The MSA then was imported into the Gblock program (71) for quality checking using the default setup and removing any misaligned regions. A phylogenetic tree was constructed using the maximum-likelihood method in IQtree (72). This program estimated the evolutionary model of sequences, and the best model, according to Akaike criterion, was GTR + I + gamma. The branch support was calculated by the bootstrap method, using 1,000 replicates. The graphic representation of the phylogeny was obtained by using iTOL web viewer (https://itol.embl.de/) (73).

### Staphylococcal enterotoxin measurement.

The 32 S. aureus strains used for the enterotoxin measurements are shown in Fig. 3A and B. These were selected based on their allocation to the different *v*Saβ islands (Table 1). If available, three strains per *v*Saβ type were used. The selected strains were cultivated on plate count agar (PCA; Becton, Dickinson, Franklin Lakes, NJ) for 24 h at 37°C, and then 3 single colonies were taken and suspended in 45 ml brain heart infusion broth (BHI; Becton, Dickinson). The inoculated broth was then incubated at 37°C for 24 h in a flask with shaking. After 24 h, the optical density of the culture was measured to check the growing of the cells (OD_480_ > 1.8). The culture was transferred to a falcon tube and centrifuged at 8,000 × *g* for 15 min at room temperature. The supernatant was then filtered through a 0.2-μm syringe filter, and the resulting filtrate was used for the downstream analysis. Quantitative analysis of SEG and SEI was performed by using an in-house sandwich quantitative ELISA. *seg* (GenBank accession no. CP001781.1) and *sei* (GenBank accession no. CP001781.1) genes from S. aureus were synthesized (Genecust) and inserted into a bacterial plasmid [isopropyl-β-d-thiogalactopyranoside (IPTG)-inducible pET22b(+) vector; Novagen, Merck] for inducible expression of recombinant SEG and SEI toxins (here used as immunogens and standards). Specific laboratory-made monoclonal antibodies were used as coating and probing biotinylated antibodies. Briefly, Biozzi mice were immunized 4 times at 3-week intervals with 10 μg of recombinant SEG or SEI toxin in alum adjuvant (intraperitoneal injection). After intravenous boost injections, hybridomas were produced by fusing spleen cells with NS1 myeloma cells, as previously described by Köhler and Milstein (74). Monoclonal antibodies were produced from hybridoma culture supernatants and further purified by protein A or protein G affinity chromatography using the AKTAxpress system (GE Healthcare, Chicago, USA).

Two separate 96-well polystyrene microtiter plates (MaxiSorp; Nunc, Roskilde, Denmark) were coated with 100 μl of monoclonal anti-SEG IgG or anti-SEI IgG (SEG41 and SEI27) at 10 μg/ml in 50 mM phosphate-buffered saline (PBS), pH 7.4, overnight at room temperature (RT), and blocked with 300 μl/well of enzyme immunoassay buffer (0.1 M PBS, pH 7.4, 1 g/liter bovine serum albumin, 0.1 g/liter sodium azide) for at least 4 h at RT. Saturated microplates were washed by 300 μl of phosphate-Tween 20 before use. A calibration curve was prepared with dilutions of SEG- and SEI-purified recombinant toxins with five concentrations between 0 and 0.3 ng SEG/ml and 0 and 2.0 ng SEI/ml, respectively (duplicate calibration points per level). Samples and recombinant standard toxins (100 μl/well) were distributed and incubated at RT for 60 min and washed three times with PBS-Tween 20, followed by addition of 100 ng/ml of biotinylated monoclonal anti-SEG or anti-SEI antibody (SEG27 and SEI26) at RT for 60 min. After extensive washing, 100 μl/well of poly-horseradish peroxidase-labeled streptavidin (dilution 1/50,000; Thermo Fisher Scientific) was used for detection at RT for 30 min and washed 5 times again. Substrate solution (100 μl/well) containing tetramethylbenzidine (TMB; Thermo Fisher Scientific, Waltham, MA) then was added for 30 min. Finally, the reaction was stopped by addition of 100 μl of H_2_SO_4_ 2 N. Absorbances were read at 450 nm on a microplate reader (SAFAS; Monaco). Quantification was performed by using a calibration curve based on the quadratic fit model. Validation data (sensitivity, specificity, and repeatability) of the above-described method are unpublished (Cécile Féraudet-Tarisse, Céline Goulard-Huet, Yacine Nia, Karine Devilliers, Dominique Marcé, Chloé Dambrune, Donatien Lefebvre, Jacques-Antoine Hennekinne, and Stéphanie Simon, unpublished data).

### Statistical analysis.

For analysis of potential correlation between production of SEG and SEI, a regression analysis was performed. First, the robust method was applied to verify the regression model and to identify outliers. Four outliers were identified and eliminated from the data set before calculating an ordinary least-square regression model.

To proof the two different levels of SEG and SEI production, a Kruskal-Wallis test was performed. For all statistical analyses, measured values under the limit of detection were taken as the value 0.

All statistical analyses were performed in Systat (version 13; Systat, Chicago, IL).

The graphical presentation of the enterotoxin data was performed using R (version 3.4.4) with the packages ggplot (75), ggsignif (76), and ggpubr (77). With these packages, the data of the enterotoxin production of the single strains were plotted in increasing order of production (means ± standard deviations) and a color given according to their relative *v*Saβ type.

### Data availability.

Sequencing data for all isolates analyzed in this study have been deposited in the NCBI GenBank database under BioProject accession number PRJNA633807. Accession numbers for individual genomes and assembly statistics are listed in Tables S2 and S3.

## Supplementary Material

Supplemental file 1
