# Supplementary material for: Staphylococcal Enterotoxin Gene Cluster: Prediction of Enterotoxin (SEG and SEI) Production and of the Source of Food Poisoning on the Basis of vSaβ Typing
Source: Appl Environ Microbiol. 2021 Feb 12;87(5):e02662-20. doi: 10.1128/AEM.02662-20 (PMC8090894; doi:10.1128/AEM.02662-20)
Supplement: Supplemental file 1 [file AEM.02662-20-s0001.pdf]

Supplementary Material

Table S1

Strain	SEG [ng/ml] +/- SD	SEI [ng/ml] +/- SD
05CEB52STA	2.59 +/- 0.46	15.25 +/- 2.94
07CEB90STA	0.9 +/- 0.10	14.34 +/- 1.38
11CEB279STA	1.55 +/- 0.14	20.63 +/- 1.97
13CEB177STA	0.80 +/- 0.11	11.18 +/- 1.85
13CEB179STA	0.99 +/- 0.11	21.03 +/- 3.77
13CEB181STA	0.78 +/- 0.13	1.86 +/- 0.92
13CEB182STA	0*	3.08 +/- 0.34
13CEB188STA	1.37 +/- 0.12	18.52 +/- 1.08
13CEB190STA	0*	2.58 +/- 0.74
13CEB323STA	3.79 +/- 1.58	61.43 +/- 10.29
13CEB327STA	1.00 +/- 0.19	2.33 +/- 1.16
15SBCL1299STA	2.42 +/- 0.58	40.8 +/- 5.23
15SBCL1397STA	2.31 +/- 0.55	36.32 +/- 3.98
15SBCL1438STA	0*	2.11 +/- 1.21
15SBCL1517STA	0.99 +/- 0.21	21.97 +/- 2.19
15SBCL1527STA	1.62 +/- 0.28	26.53 +/- 0.94
17SBCL08STA	1.23 +/- 0.06	11.24 +/- 0.39
17SBCL13STA	1.42 +/- 0.14	14.89 +/- 0.98
18SBCL669	1.77 +/-0.01	3.85 +/- 0.99
18SBCL670	0*	1.15 +/- 0.47
18SBCL672	0*	10.77 +/- 1.22
18SBCL673	2.04 +/- 0.33	40.58 +/- 9.03
18SBCL674	2.11 +/- 0.21	37.66 +/- 3.69
18SBCL675	0.26 +/- 0.01	3.33 +/- 0.33
18SBCL677	1.34 +/- 0.10	22.65 +/- 3.13
18SBCL678	0*	2.84 +/- 0.28
18SBCL679	1.73 +/- 0.12	29.05 +/- 3.48
G19F	1.05 +/- 0.13	16.29 +/- 0.76
G68P	1.08 +/- 0.10	1.403 +/- 0.64
M2323	0*	1.06 +/- 0.17
M3783	0*	1.57 +/- 0.22
Mu50	4.26 +/- 0.78	35.63 +/- 3.02

**under the limit of detection*

Table S2

Strain name	Bio Project	Accession Nr.
05CEB52STA	PRJEB36867**	ERS4338875
07CEB90STA	PRJEB36867**	ERS4338881
07CEB94STA	PRJEB36867**	ERS4338884
11CEB145STA	PRJEB36867**	ERS4338891
11CEB277STA	PRJEB36867**	ERS4338897
11CEB279STA	PRJEB36867**	ERS4338899
13CEB177STA	PRJEB36867**	ERS4338908
13CEB178STA	PRJEB36867**	ERS4338909
13CEB179STA	PRJEB36867**	ERS4338910
13CEB181STA	PRJEB36867**	ERS4338911
13CEB182STA	PRJEB36867**	ERS4338912
13CEB188STA	PRJEB36867**	ERS4338914
13CEB190STA	PRJEB36867**	ERS4338915
13CEB191STA	PRJEB36867**	ERS4338916
13CEB312STA	PRJEB36867**	ERS4338928
13CEB313STA	PRJEB36867**	ERS4338929
13CEB317STA	PRJEB36867**	ERS4338933
13CEB318STA	PRJEB36867**	ERS4338934
13CEB323STA	PRJEB36867**	ERS4338938
13CEB327STA	PRJEB36867**	ERS4338941
13CEB328STA	PRJEB36867**	ERS4338942
13CEB329STA	PRJEB36867**	ERS4338943
15SBCL1292STA	PRJEB36867**	ERS4338961
15SBCL1299STA	PRJEB36867**	ERS4338962
15SBCL1397STA	PRJEB36867**	ERS4338965
15SBCL1409STA	PRJEB36867**	ERS4338967
15SBCL1428STA	PRJEB36867**	ERS4338968
15SBCL1438STA	PRJEB36867**	ERS4338971
15SBCL1507STA	PRJEB36867**	ERS4338973
15SBCL1517STA	PRJEB36867**	ERS4338975
15SBCL1527STA	PRJEB36867**	ERS4338977
15SBCL1550STA	PRJEB36867**	ERS4338980
17SBCL08STA	PRJEB36867**	ERS4338988
17SBCL09STA	PRJEB36867**	ERS4338989
17SBCL13STA	PRJEB36867**	ERS4338990
17SBCL202STA	PRJEB36867**	ERS4338992
17SBCL208STA	PRJEB36867**	ERS4338993
17SBCL214STA	PRJEB36867**	ERS4338994
17SBCL220STA	PRJEB36867**	ERS4338995
17SBCL225STA	PRJEB36867**	ERS4338997
17SBCL580STA	PRJEB36867**	ERS4339000

17SBCL585STA	PRJEB36867**	ERS4339001
18 SBCL671*	PRJNA633807	JABUAB000000000
18 SBCL 676*	PRJNA633807	JABUAA000000000
18 SBCL 680*	PRJNA633807	JABTZZ000000000
18 SBCL667*	PRJNA633807	JABTZY000000000
18SBCL669*	PRJNA633807	JABTZX000000000
18SBCL670*	PRJNA633807	JABTZW000000000
18SBCL672*	PRJNA633807	JABTZV000000000
18SBCL673*	PRJNA633807	JABTZY000000000
18SBCL674*	PRJNA633807	JABTZX000000000
18SBCL675*	PRJNA633807	JABTZU000000000
18SBCL677*	PRJNA633807	JABTZT000000000
18SBCL678*	PRJNA633807	JABTZS000000000
18SBCL679*	PRJNA633807	JABTZR000000000
502A	-	CP007454.1
ATCC25923	-	CP009361.1
G11F	PRJNA531079	SZYL00000000
G19F	PRJNA531079	SZYN00000000
G68P	PRJNA531079	SZYR00000000
GN3	-	AP017891
KS90*	PRJNA633807	JABTZQ000000000
M1280	PRJNA531079	SZYW00000000
M1655	PRJNA531079	SZYX00000000
M2323	PRJNA531079	SZYZ00000000
M2682	PRJNA531079	SZZB00000000
M2839	PRJNA531079	SZZC00000000
M3783	PRJNA531079	SZZE00000000
MRSA252	-	BX571856
Mu50	-	BA000017
N315	-	BA000018
NZAK3	-	LT009690
RF122	-	AJ938182
ST288	-	FFXA01000001
USA600	-	CP006044

*Strains sequenced in this study

** Data only available on Ena database (www.ebi.ac.uk)

Table S3

Strain	Coverage [X]	Nr. of contigs	Contig N50 [bp]	Cds
18 SBCL667	325	33	2,696,853	2487
18SBCL669	360	45	2,739,638	2263
18SBCL670	279	69	2,741,546	2590
18 SBCL671	278	49	2,805,767	2617
18SBCL672	399	19	2,737,181	2395
18SBCL673	267	17	2,751,056	2281
18SBCL674	234	41	2,748,718	2293
18SBCL675	245	19	2,829,665	2582
18 SBCL676	296	23	2,719,997	2298
18SBCL677	275	60	2,723,142	2298
18SBCL678	305	126	2,912,510	2527
18SBCL679	203	12	2,719,626	2376
18 SBCL680	336	65	2,691,599	2488
KS90	380	84	2,817,361	-

Statistical analysis data

Robust linear regression for verify the regression model and to identify outliers

**Dependent Variable**	MW_I
**No. of cases**	32
**No. of Regressors**	1

Least Median of Squares (LMS) Regression

**Method of Estimation**	Exhaustive search

**Number of Subsamples**	496

LMS Parameter Estimates

Effect	Coefficient
**CONSTANT**	0.8590
**MW_G**	15.5887

**Scale Estimates**	4.7558

**Cutoff Point**	3.0000
**Number of Outliers Detected**	4

**Robust R-Square**	0.9648

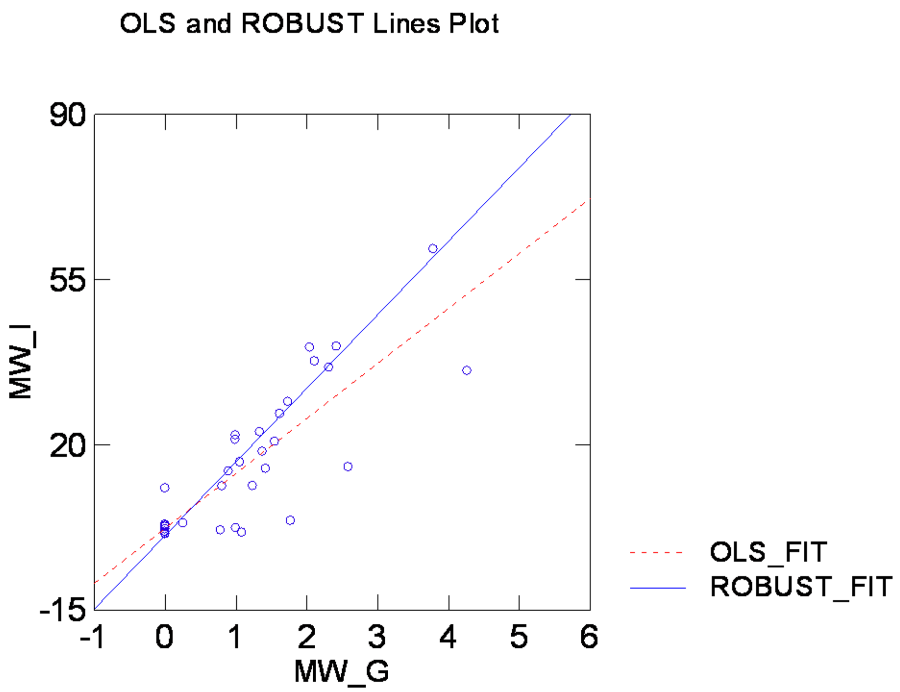

Figure S1: Ordinary least square and robust lines plot

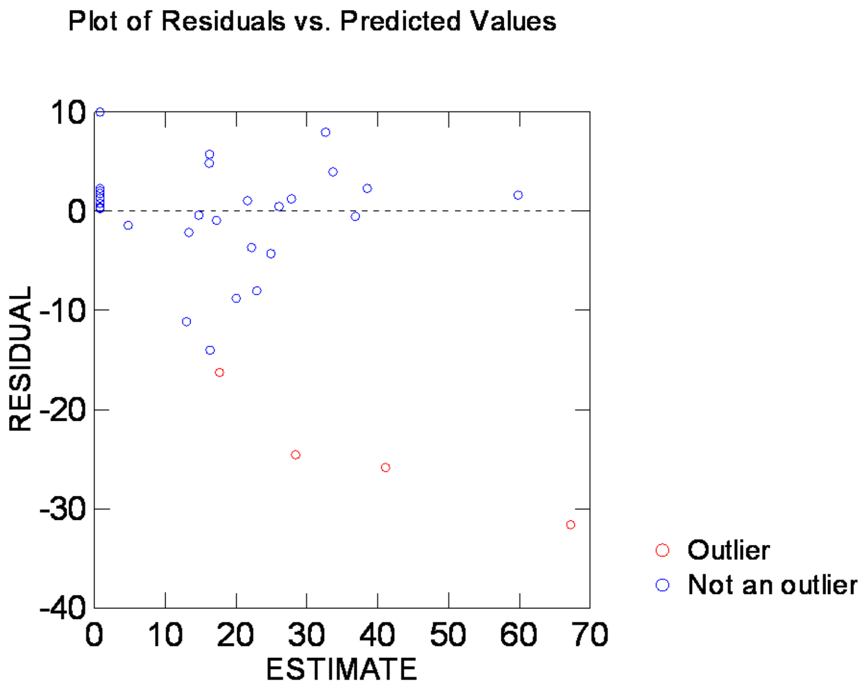

Figure S2: Plot of residuals vs. predicted values from the Robust linear regression

Ordinary least square regression analysis without outliers

**Dependent Variable**	MW_I
**N**	28
**Multiple R**	0.9400
**Squared Multiple R**	0.8837
**Adjusted Squared Multiple R**	0.8792
**Standard Error of Estimate**	5.4078

Regression Coefficients B = $(X'X)^{-1}X'Y$

Effect	Coefficient	Standard Error	Std. Coefficient	Tolerance	t	p-Value
**CONSTANT**	0.6346	1.5529	0.0000	.	0.4086	0.6862
**MW_G**	15.4884	1.1020	0.9400	1.0000	14.0550	0.0000

Analysis of Variance

Source	SS	df	Mean Squares	F-Ratio	p-Value
**Regression**	5'777.0610	1	5'777.0610	197.5428	0.0000
**Residual**	760.3596	26	29.2446		

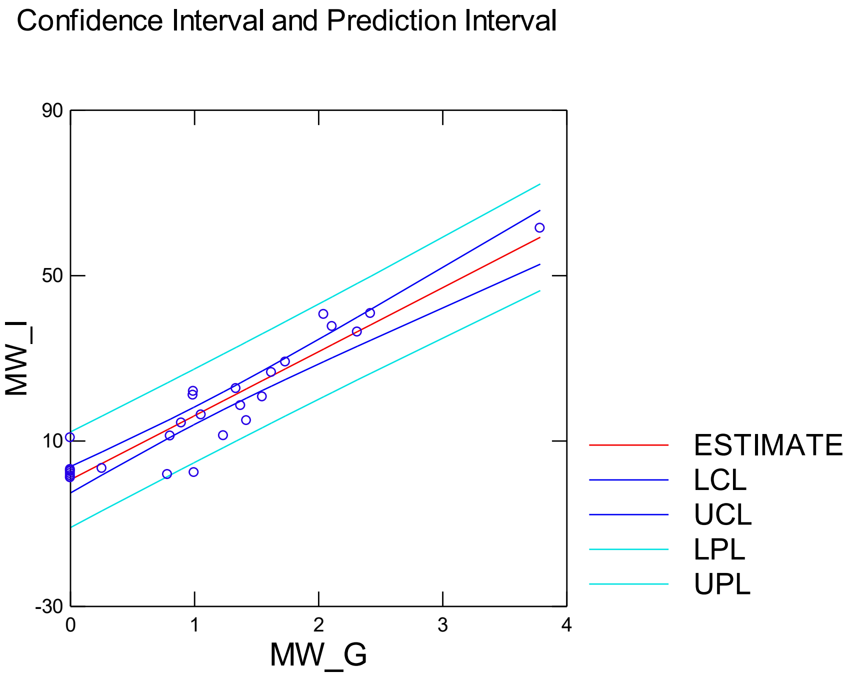

Figure S3: Confidence interval and prediction interval of the ordinary least square regression analysis without outliers

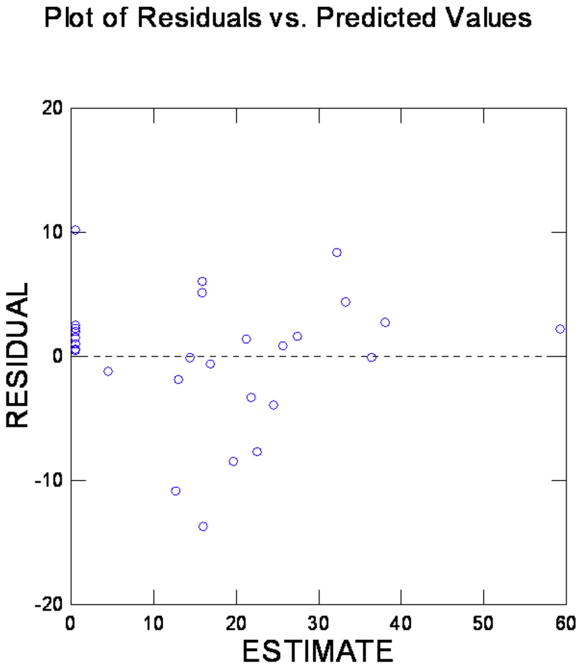

Figure S4: Plot of residuals vs. predicted values from ordinary least square regression analysis without outliers
